# Role of miR-17 Family in the Negative Feedback Loop of Bone Morphogenetic Protein Signaling in Neuron

**DOI:** 10.1371/journal.pone.0083067

**Published:** 2013-12-11

**Authors:** Qi Sun, Susu Mao, Hanqin Li, Ke Zen, Chen-Yu Zhang, Liang Li

**Affiliations:** 1 Jiangsu Engineering Research Center for microRNA Biology and Biotechnology, State Key Laboratory of Pharmaceutical Biotechnology, School of Life Sciences, Nanjing University, Nanjing, China; 2 Department of Virology, University of California School of Public Health, Berkeley, California, United States of America; University of Turin, Italy

## Abstract

Bone morphogenetic protein (BMP) signaling is active in many tissues including the central nervous system, in which it regulates cell proliferation, differentiation and maturation. The modulation of BMP pathway is crucial since abnormality of BMP signaling may cause cellular malfunction such as apoptosis. There are evidences indicating that miR-17 family is involved in the BMP signaling. In the present study, we demonstrated that BMP2 stimulation directly increased the transcription of miR-17-92 and miR-106b-25 cluster via Smad activation, which leads to the up-regulation of mature miR-17/20a/93. In addition, we provided evidence that BMP2 activation repressed BMPRII expression through modulating miR-17 family in primary neurons. Furthermore, we proved that such negative regulation protected neurons from apoptosis induced by abnormal BMP signaling. Taken together, these results suggest a regulatory pathway of BMP-miR-17 family-BMPRII, which consist a negative feedback loop that balances BMP signaling and maintains cell homeostasis in neurons.

## Introduction

BMPs (Bone Morphogenetic Proteins) are a large subclass of the TGF-β (Transforming Growth Factor-β) super family, which have crucial roles in many tissues as well as neural system [1,2]. The signaling pathway includes BMPs, BMPRs (Bone Morphogenetic Protein Receptors) and Smads, in which particular Smads are dedicated to different ligands, with R-Smad (Smad1, 5 and 8) and Co-Smad (Smad4) mediating signals from special members of the BMP subfamily [3,4]. There are substantial evidences that BMP signaling plays a crucial role in the neural development including proliferation, differentiation and maturation [5–7]. For example, BMP promotes the astroglial lineage commitment during the neural differentiation [8,9]. In addition, activation of Smad1 pathway through BMP2 or 4 facilitates the axonal growth in adult sensory neurons [10,11]. However, it is also reported that aberrant activation of BMP signaling can cause neuronal dysfunction which may lead to further disorders [12,13]. Therefore, it is of great significance to avoid the abnormality of BMP signaling in nervous system.

MiRNAs are approximately 21-nucleotide small RNAs that are derived from hairpin precursors, which repress protein expression by targeting 3’-UTR (3’-untranslated region) of mRNAs [14]. The miR-17 family consists of six members (miR-17, miR-20a, miR-20b, miR-93, miR-106a and miR-106b), which distribute in three genome clusters [15]. Unlike the miR-17-92 and miR-106b-25 cluster, which are both abundantly expressed in many sorts of tissues, the miR-106a-363 cluster is undetectable or unexpressed in most of the tissues [16,17]. There are several reports that miR-17 family functions in nervous system [18–20]. Recently, it is reported that interleukin-6 modulates the expression of BMPRII in endothelial cells through miR-17-92 pathway [21]. Furthermore, there is also evidence that BMPs can increase the miR-17-92 expression in cardiac progenitor cells [22]. Based on these studies, we hypothesize that in neuron there may be a negative feedback in BMP-miR-17 family-BMPRII circle, which helps to maintain BMP signaling in a proper range under normal physiological conditions.

To verify this notion, we first examined the expression of miR-17 family in primary neurons after BMP stimulation and found that BMP2 increased miR-17-92 and miR-106b-25 cluster transcription through the activation of Smads, which directly bound to the promoter region of those clusters. Further investigation showed that BMP2 mediated up-regulation of those miRNAs lead to the repression of BMPRII in neuronal cells. Moreover, we also demonstrated that the negative feedback in BMP-miR-17 family-BMPRII circle can alleviate neuronal apoptosis induced by over-stimulation of BMP2. Taken together, our data suggest that miR-17 family functions in a feedback loop of BMP signaling pathway in primary neuron, which helps to maintain cellular homeostasis.

## Materials and Methods

### Animals

Animal studies were conducted in strict accordance with the principles and procedures approved by the Committee on the Ethics of Animal Experiments of Nanjing University. C57BL/6 Mice were housed under a 12-h light/12-h dark cycle and fed autoclaved water and laboratory rodent chow. The mother mice were sacrificed by cervical dislocation and brains were dissected out from embryos at E15.5.

### Cell Culture

Primary mouse cortical neuron cultures from embryonic day 15.5 (E15.5) C57BL/6 pregnant mice were obtained and maintained as previously described [23]. In brief, neocortices from fetal mice were dissociated and plated onto six-well plates pre-coated with Poly-D-Lysine (Sigma) at a density of 1×10^5^ per square centimeter for the neuron cultures, and maintained in Neurobasal Medium supplemented with 2%(v/v) B27 Supplement, 1 mM-glutamine and 1% (v/v) penicillin/streptomycin (all reagents provided by Gibco-Invitrogen). The cells were cultured for 3 days before transfection. Human neuroblastoma SH-SY5Y cells were purchased from ATCC. The cells were maintained in DMEM supplemented with 10 % (v/v) FBS and 1% (v/v) penicillin/streptomycin (all reagents provided by Gibco-Invitrogen) at 37 °C in an atmosphere containing 5 % CO_2_. BMP2 (Prospec) was used in the cell culture medium with the indicated concentration.

### Quantitative Reverse Transcriptase-PCR (qRT-PCR)

qRT-PCR was performed using TaqMan microRNA probes (Applied Biosystems), as previously described [24]. Briefly, Total RNAs were isolated using TRizol (Invitrogen) and reverse transcribed to produce cDNA using AMV reverse transcriptase (TaKaRa) and stem-loop RT primers (Applied Biosystems). Real-time PCR was performed using a TaqMan PCR kit and an Applied Biosystems 7300 Sequence Detection System (Applied Biosystems). All of the reactions were run in triplicates. mRNA level was normalized to glyceraldehyde-3-phosphate dehydrogenase (GAPDH) and microRNA level was normalized to U6 snRNA. Primer sets for Id1, pri-miR-221, pri-miR-17-92 and pri-miR-106b-25 have been published previously [25–27] and showed as followed:

Id-1: Forward: 5’-AACCGCAAGGTGAGCAAGGTGG-3’; Reverse: 5’-ACGCATGCCGCCTCGGC-3’.
Pri-miR-221: Forward: 5’- GCAACTGCTGCACAAATACC-3’; Reverse: 5’-TTGATAAAGGGCTGCTGGAC-3’;
Pri-miR-17-92: Forward: 5’-TTGGAACTTCTGGCTATTGGCTCCTC-3’; Reverse: 5’-CCAAGGTGAGGTTCACTTTATTCCTGC-3’;
Pri-miR-106-25: Forward: 5’- AAAGGCTGCTTGCTGCTTGAATCC-3’; Reverse: 5’-ACTAAGGTCCAAGAGGGGAGGACAG-3’

### Plasmid construction

To test the binding of miR-17 family to its target gene BMPRII, the entire segment of mouse BMPRII 3’-untranslated region (3’-UTR), containing a presumed miR-17 family binding site (seed sequence, 5’-GCACUUU-3’), was inserted into the pMIR-REPORT plasmid (Applied Biosystems). To test the binding specificity, we mutated the binding sequence of BMPRII from 5’-GCACU-3’ to 5’-CGUGA-3’.

For the promoter assay experiments, the different fragments of the miR-106b-25 cluster promoter were cloned into the XhoI-HindIII-digested pGL3 basic vector (Promega). -0.6-(mut)-Luc was generated as indicated.

The miR-106b-25 promoter fragments were PCR-amplified using following primers:
m106b-25(-2.0kb)XhoI F: 5’-GATCTCGAGCTGAACGGCTTCTGTTGTAAAT-3’;
m106b-25(-0.6kb)XhoI F: 5’-GATCTCGAGTGCCACATCACGACCAC-3’;
m106b-25(-0.2kb)XhoI F: 5’-ATACTCGAGCCCTCAAGCCCCTTGTCATTC-3’;
m106b-25(-0kb)HindIII R: 5’-ATAAAGCTTGGCAGGACTTGAAGGGCTCA-3’;

For BMP-induced Smad activity assay, we cloned the fragment of ID1 promoter into the XhoI-HindIII-digested pGL3 basic vector (Promega).

ID1(-1585)XhoI F: 5'-CCGCTCGAGGGGTCTCTGAGACCTAACTGTTCGCCCCCAGT-3'
ID1(+88)HindIII R:5'-CCCAAGCTTGGGGATCCTGAGAACAGGCGGAGGGGAGCGGAG-3'[28].

### Cell transfection and luciferase reporter assay

Primary neurons or SH-SY5Ycells were seeded on six-well plates and were transfected with miR-17/miR-93 mimics (100 pmol, Genepharma), miR-17/miR-93 inhibitors (100 pmol, Genepharma) , miR-17 family sponge (4 ug, Addgene, #21970) or Smad4 siRNA (100 pmol, Invitrogen, 5’-UACAAAGACCGCGUGGUCACUAAGG-3’) using Lipofectamine 2000 (Invitrogen) according to the manufacturer’s instructions. Cells were harvested 24 h after transfection for qRT-PCR and 48 h for protein analysis.

For the luciferase reporter assays, 1.0 ug pGL3 promoter plasmid or 0.8 ug pMIR-REPORT luciferase reporter plasmid, 0.1 ug β-galactosidase (β-gal) expression vector (Applied Biosystems), and where indicated 20 pmol miR-17/miR-93 mimics, miR-17/miR-93 inhibitors, or scrambled oligonucleotides were transfected into SH-SY5Y cells or primary neuron cells cultured in 24-well plates using Lipofectamine 2000 (Invitrogen), according to the manufacturer’s instruction. The β-gal vector was used as a transfection control. Forty-eight hours after transfection, alternatively followed by BMP stimulation, the cells were assayed using a luciferase assay kit (Promega).

### Chromatin immunoprecipitation (ChIP)

ChIP assay was performed using a ChIP assay kit (Upstate). With or without BMP2 stimulation (10 ng/ml, 6h), soluble chromatin was prepared from primary cortical neurons and incubated with anti-phospho-Smad1/5 antibody (#9516, Cell Signaling), anti-Smad4 antibody (#9515, Cell Signaling), or mouse IgG as negative control. miR-106b-25 promoter primers: 5’-CCTCCAGGCAGTCCCGTCAG-3’ and 5’-GGGTTCAACTTTCACCGTGT-3’

TCT-3’. Negative control primer (>2 kb upstream): 5’-ATATTAAAGGCACGCACCACC-3’ and 5’-ATAGCAAGCACCAGCAGAGC-3’. As a positive control for Smad binding site, PCR primers for mouse ID1 promoter were used: 5’-AGCGGAGAATGCTCCAGCCCA-3’ and 5’-AGGCCTCCGAGCAAGCTCTCCCT-3’[28].

### Western blotting

Protein samples were quantified using a BCA kit (Thermo Scientific, Rockford, IL, USA). 50 μg protein of each sample was separated using 10% SDS-PAGE and transferred onto PVDF Western Blotting Membranes (Roche Diagnostics). The membranes were incubated with primary antibodies overnight at 4°C. The primary antibodies were used as follows: anti-BMPRII (#612292, BD), anti-PTEN (#9552, Cell Signaling), anti-AKT (#9272, Cell Signaling), anti-phospho-Akt (#4056, Cell Signaling), anti-Asp175-cleaved-caspase3 (#9664, Cell Signaling), anti-caspase3 (#9662, Cell Signaling) and anti-GAPDH (sc-32233, Santa Cruz). Horseradish peroxidase anti-mouse and anti-rabbit (Santa Cruz Biotechnology) were used as secondary antibodies. The signal was detected by the super-signal-enhanced chemiluminescence system (Pierce, Rockford, IL, USA). 

### TUNEL Assay

Briefly, primary cortical neurons were transfected with miRNAs mimics or sponge at DIV 3 (days in vitro), followed with BMP2 (50 ng/ml, Prospec) stimulation for 5 days. After that the cells were harvested and washed with PBS, and then fixed and permeabilized, followed by TUNEL labeling using a One Step TUNEL Apoptosis Assay Kit (KGA7052, KeyGEN Biotech) as instructed. The percentage of apoptotic cells was estimated by the percentage of cells with positive DAB staining of five randomly selected fields in each slide.

### Statistical Analysis

All images of Western blots were representatives of at least three independent experiments. qRT-PCR and luciferase reporter assays were performed in triplicates. Data shown are the mean ± SD for three or more independent experiments. Differences were considered statistically significant at *p < 0.05 or **p < 0.01, assessed using the Student’s t test.

## Results

### BMP2 up-regulates miR-17 family in miR-17-92 and miR-106b-25 cluster in a Smad dependent manner

**Figure 1:**
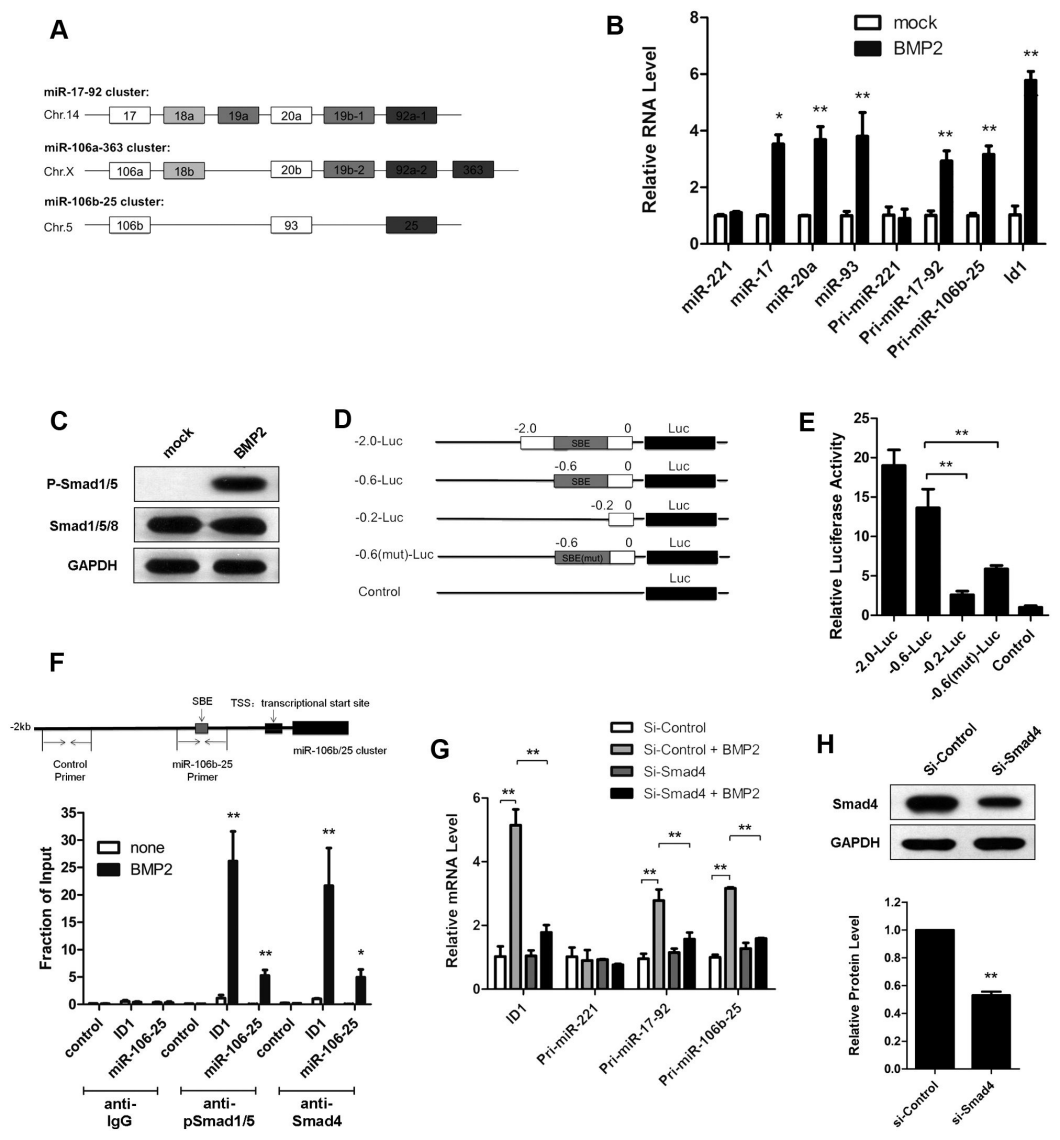
BMP2 up-regulates miR-17 family expression in miR-17-92 and miR-106b-25 cluster through Smad activation. (**A**) Schematic diagram showing that miR-17 family located in mouse miR-17-92 (Chr.14), miR-106a-363 (Chr.X) and miR-106b-25 (Chr.5) cluster. The color code identifies miRNAs with the same seed sequence. (**B**) qRT-PCR analysis of miR-17/20a/93 expression in primary cortical neurons (miR-221 served as negative control; Id1 served as positive control; U6 snRNA was used as internal control). Cells were subjected to RNA Analysis 6 h after the stimulation with BMP2. *p< 0.05, **p < 0.01, vs mock. (**C**) Protein level of phosphorylated Smad1/5 in primary cortical neuron treated with or without BMP2 (10 ng/ml) stimulation for 6 h. (**D**) Schematic diagram of pGL3 constructs containing different fragments of the promoter of miR-106b-25 cluster. A Smad-binding element (SBE) upstream of the miR-106b-25 cluster is indicated. (**E**) Promoter assay revealed the functional region of miR-106b-25 promoter responded to BMP2. Relative luciferase activity of different groups of cells is shown. SH-SY5Y cells were transfected with one of those reporter constructs (indicated in D) followed with the treatment of BMP2 (10 ng/ml) for 6 h. An empty pGL3 basic vector was used as a control. **p< 0.01, vs -0.6-Luc transfected cells. (**F**) ChIP analysis proved a Smad-binding element existed in miR-106b-25 promoter. Upper: Schematic representation of the genomic region upstream of the miR-106b-25 cluster. Bottom: ChIP with an anti-pSmad1/5 antibody or anti-Smad4 antibody on lysates from primary cortical neurons pre-treated with or without BMP2 (10 ng/ml) for 6 h. Normal mouse immunoglobulin G was used as a negative control. qPCR was then performed to measure enrichment of DNA fragment by primers, such as control, miR-106b-25, or ID1 promoter region. The data were plotted as relative enrichment to input. *p < 0.05, **p < 0.01, vs cells without treatment. (**G**) qRT-PCR analysis of pri-miRNAs in primary cortical neurons transfected with control siRNAs (Si-Control) or siRNAs against Smad4 (Si-Smad4), followed with or without BMP (10 ng/ml) stimulation for 6 h. GAPDH mRNA was used as an internal control. **p < 0.01. (**H**) Protein analysis of endogenous Smad4 in primary cortical neurons transfected with Si-Control or Si-Smad4, followed by BMP (10ng/ml) stimulation for 6 h. **p < 0.01, vs Si-Control. Data shown are the mean ± SD for three independent experiments.

There are six miRNA members (miR-17, miR-20a, miR-20b, miR-93, miR-106a and miR-106b) in miR-17 family, which locate in three separate genome clusters (Figure 1A). It is reported that miR-106a-363 cluster is undetectable in almost all cell types including brain [17]. Therefore, we mainly analyzed the expression of miR-17 family members in miR-17-92 and miR-106b-25 cluster.

Here we examined the expression level of the miR-17/20a/93 in primary cortical neurons activated with BMP2 (10 ng/ml) and showed that all those miRNAs increased after the treatment (Figure 1B). Since those increased miRNAs are located in miR-17-92 and miR-106b-25 cluster, we further examined primary transcripts of these two clusters. We demonstrated that pri-miR-17-92 and pri-miR-106b-25 were both up-regulated (~3 folds) upon BMP2 treatment, similarly to mature miR-17/20a/93, suggesting that BMP2 stimulation increased the transcription of these two clusters (Figure 1B). These data indicate that BMP2 treatment up-regulates the transcription of miR-17-92 and miR-106b-25 cluster, which results in the increased level of mature miR-17/20a/93 in primary cortical neurons.

Most transcriptional regulations of BMP are carried out via Smads, which are also activated in primary neurons followed with BMP2 treatment (Figure 1C). Previous studies revealed a binding site of Smads in the promoter of miR-17-92 cluster [22]. Therefore, we further investigated whether they also regulated the transcription of miR-106b-25 cluster. To determine the functional region of miR-106b-25 promoter responded to BMP2, various fragments [-2.0-Luc (-1973bp to +44bp), -0.6-Luc (-562bp to +44bp) and -0.2-Luc (-177bp to +44bp)] of miR-106b-25 promoter were evaluated, which indicated that region (-0.6 to -0.2 kb) of miR-106b-25 promoter is essential for the response to BMP2 (Figure 1D-E). Through bioinformatics approaches we uncovered a conserved Smad-binding element (SBE, 5’-GTCT-3’, -429bp to -426bp) in miR-106b-25 promoter (Figure 1D and Figure S1) [29]. To clarify whether this putative SBE was involved in the miRNA induction, we further generated a mutated SBE plasmid (-0.6(mut)-Luc, 5’-CATA-3’) and found that its response to BMP2 induction was strongly compromised, indicating this SBE as a critical regulative target of BMP/Smad pathway (Figure 1E).

In addition, our chromatin immunoprecipitation (ChIP) assay in primary neurons subjected to BMP2 stimulation also indicated a direct binding between Smad1/5-Smad4 complex and the promoter of miRNA-106b-25 (Figure 1F). Genomic DNA fragments associated with phospho-Smad1/5 or Smad4 were immunoprecipitated with appropriate antibodies (mouse IgGs were used as negative control), followed by qPCR amplification with either a primer set recognizing the region containing the SBE (miR-106b-25 promoter primers, Figure 1F, top) or a control primer set that recognizes sequence ~2 kb upstream of the SBE (control primers, Figure 1F, top). Another primer set recognizing the ID1 promoter was used as positive control. The results demonstrated that both miR-106b-25 promoter and ID1 promoter showed positive signals for binding to Smad1/5-Smad4 complex after BMP2 stimulation. Furthermore, the induction of miR-17-92 and miR-106b-25 cluster by BMP2 was significantly inhibited after we blocked the BMP signaling pathway by repressing Co-Smad4 expression (~50% knockdown) with siRNA (Figure 1G-H). These results provide strong evidence that BMP directly induces miR-17-92 and miR-106b-25 transcription in Smad-dependent manner.

### Repression of BMPRII translation by miR-17/93

**Figure 2:**
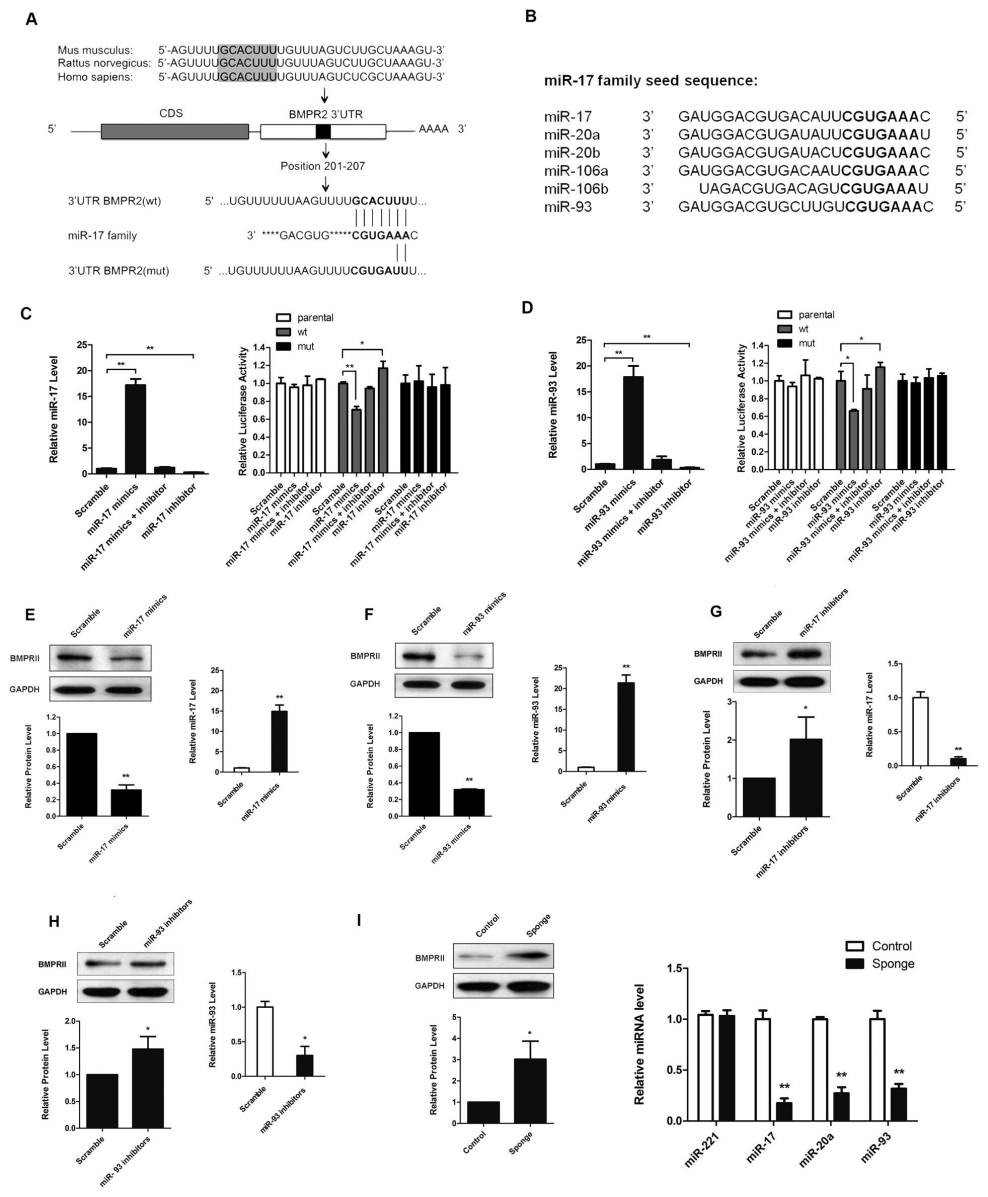
miR-17/93 represses BMPRII expression. (**A**) Phylogenetic sequence alignment of miR-17 family seed sequence in wild-type (wt) and mutant (mut) BMPRII 3’-UTR. Luciferase reporters carrying wild-type or mutant BMPRII 3’-UTR were co-transfected into SH-SY5Y cells along with the indicated oligonucleotides. Cells were maintained for 48 hours before luciferase activity was determined. (**B**) Mature miRNA sequences of miR-17 family with seed sequence capital highlighted. (**C**-**D**) RNA analysis (left) and luciferase reporter activity (right) of different groups of SH-SY5Y cells transfected with miR-17/93 mimics or/and miR-17/93 inhibitors. *p< 0.05, **p < 0.01, vs Scramble. (**E**-**I**) Protein analysis of BMPRII (left) and miRNA levels (right) in primary neuron transfected with miR-17/93 mimics, miR-17/93 inhibitors or miR-17 family sponge. *p< 0.05, **p < 0.01, vs Scramble/Control. Data shown are the mean ± SD for three independent experiments.

There is evidence that BMPRII is regulated by miR-17/20a in neurons [21]. We retrieved an evolutionarily conserved site in 3’-UTR of BMPRII mRNA that is complementary to the seed sequence of miR-17 family (Figure 2A-B). Here we further tested whether other members of miR-17 family can modulate the expression of BMPRII in neuronal cells. We demonstrated that the relative expression levels of miR-17/93 in SH-SY5Y cells were significantly increased (~17 folds) after the transfection of miR-17/93 mimics, while miR-17/93 inhibitors markedly blocked such increase. Furthermore, these inhibitors even decreased endogenous miR-17/93 level in SH-SY5Y cells when used alone (~30%, Figure 2C-D). After being normalized toβ-gal activity, luciferase assay in SH-SY5Y cells revealed that over-expression of miR-17/93 significantly reduced translational efficiency (~30%) of reporter gene tagged with 3’-UTR of BMPRII, while this reduction was abolished upon miR-17/93 inhibitors. Meanwhile, transfection of miR-17/93 inhibitors alone resulted in a ~20% increase in luciferase reporter activity compared with the scrambled oligonucleotides-transfected cells (Figure 2C-D). After mutating the seed sequence in 3’-UTR of BMPRII, the inhibitory effect of miR-17/93 on luciferase reporter activity was largely compromised (Figure 2C-D).

Next, we further evaluated the modulating effect of miR-17/93 and miR-17 family sponge on BMPRII expression in primary neurons. Over-expression of miR-17/93 mimics not only increased endogenous miR-17/93 level (~20 folds) but also repressed the protein expression of BMPRII compared with scrambles (~70%, Figure 2E-F). On the contrary, inhibition of miR-17/93(~70%) caused the BMPRII level to increase by 1.5~2 folds (Figure 2G-H). In addition, transfection of miR-17 family sponge decreased miR-17/20a/93 level simultaneously while it had little effect on miR-221 level (Figure 2I). In the meantime, miR-17 family sponge also increased the protein level of BMPRII by ~3 folds (Figure 2I) which indicated that miR-17 family sponge is more efficient than specific miRNA inhibitors in modulating BMPRII expression. Together, these data suggest that BMPRII is a target gene of miR-17 family in neuronal cells.

### BMP signaling negatively regulates BMPRII expression via up-regulated miR-17

**Figure 3:**
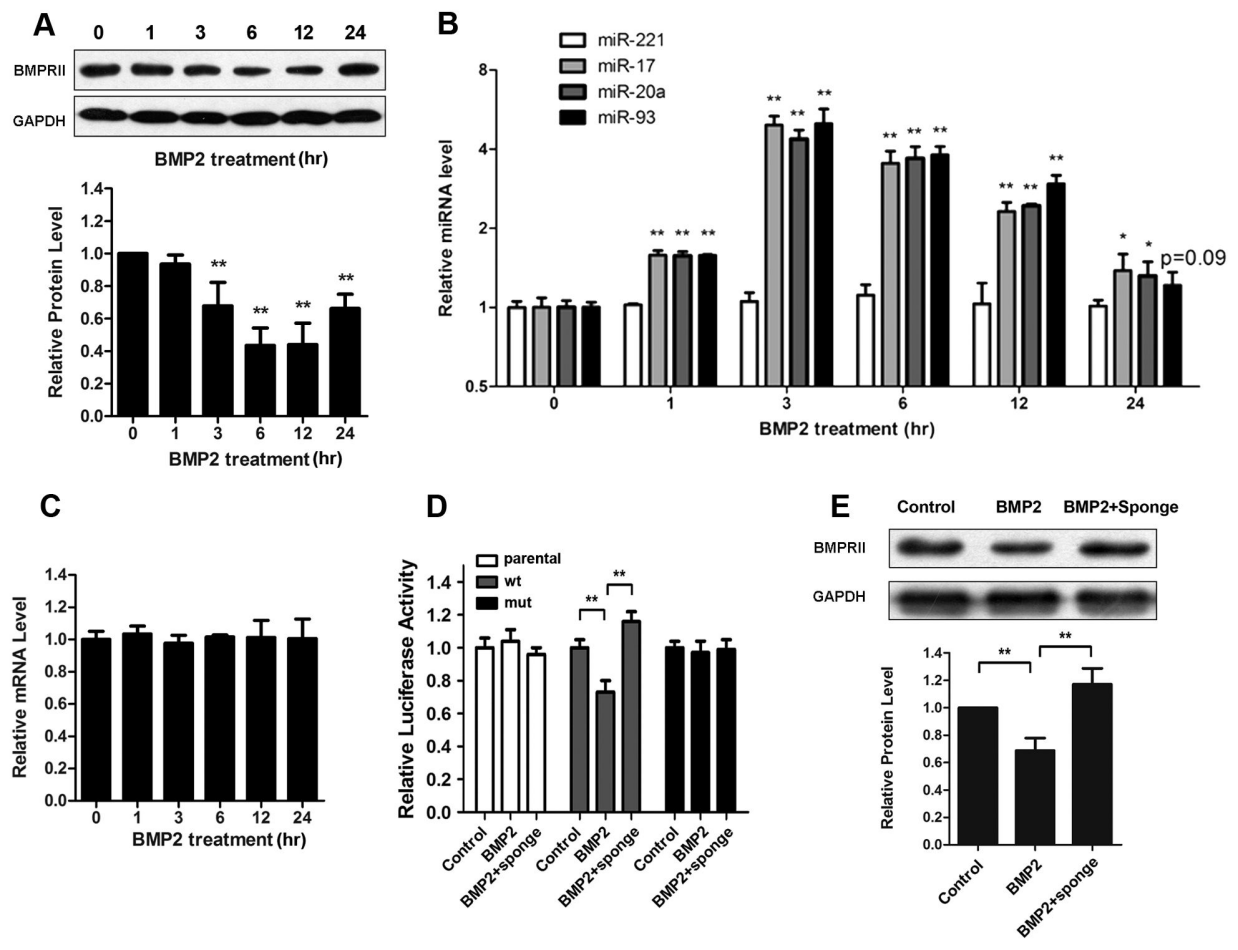
BMP2 negatively regulates BMPRII protein level through induction of miR-17 family. (**A**) Time course of BMPRII protein levels in primary cortical neurons under BMP2 (10 ng/ml) treatment: representative Western blot (upper panel) and quantitative analysis of BMPRII level (bottom panel). **p < 0.01, vs 0 h BMP2. (**B**) Time course of miRNA expression in primary cortical neurons under the treatment of BMP2 (10ng/ml). *p< 0.05, **p < 0.01, vs 0 h BMP2. (**C**) Time course of BMPRII mRNA levels in primary cortical neurons under BMP2 (10ng/ml) treatment. (**D**) Luciferase reporter activity of different groups of SH-SY5Y cells. **p < 0.01, vs Control. (**E**) Protein analysis of BMPRII in different groups of primary culture: representative Western blot (upper panel) and quantitative analysis of BMPRII level (bottom panel). **p < 0.01, vs Control. Data shown are the mean ± SD for three independent experiments.

We have demonstrated that BMP2 increased miR-17/20a/93 level while these miRNAs repressed BMPRII translation. Here we further investigated the effect of BMP signaling on BMPRII expression in primary culture. We found that BMP2 treatment decreased BMPRII protein level, which started to fall at 3 h (~30% reduction) and dramatically down-regulated during 6-12 h stage (~60% reduction) (Figure 3A). Meanwhile, we observed an increase of miR-17/20a/93 level following BMP2 stimulation, which reached peak during 3-6 h stage (~5 folds) and then gradually decreased after 12 h (Figure 3B). These data showed a negative correlation between BMPRII protein and mature miR-17/20a/93 level in primary neurons after BMP2 activation. Besides, the corresponding mRNA levels of BMPRII were essentially stable throughout the treatment indicating the translational inhibition of BMPRII in which up-regulated miR-17 may be involved (Figure 3C).

To further elucidate the role of miR-17 family in BMP2 mediated BMPRII repression, we conducted luciferase assay and found that BMP2 activation significantly reduced the expression of reporter gene tagged with 3’-UTR of BMPRII while pretreatment with miR-17 family sponge blocked such effect (Figure 3D). In addition, the response to BMP2 was also compromised after we mutated the seed sequence in 3’-UTR of BMPRII (Figure 3D). Furthermore, protein analysis also revealed that level of BMPRII down-regulated upon BMP2 stimulation while miR-17 family sponge rescued such decrease in neurons, which is consistent with the results from luciferase assay in SH-SY5Y cells (Figure 3E). These results indicate that the increased level of miR-17 family markedly contributes to the BMP2 mediated BMPRII repression.

Taken together, we have demonstrated a negative feedback loop of BMP-miR-17 family-BMPRII in neuronal cells.

### MiR-17 modulates Smad activity after BMP2 stimulation

**Figure 4:**
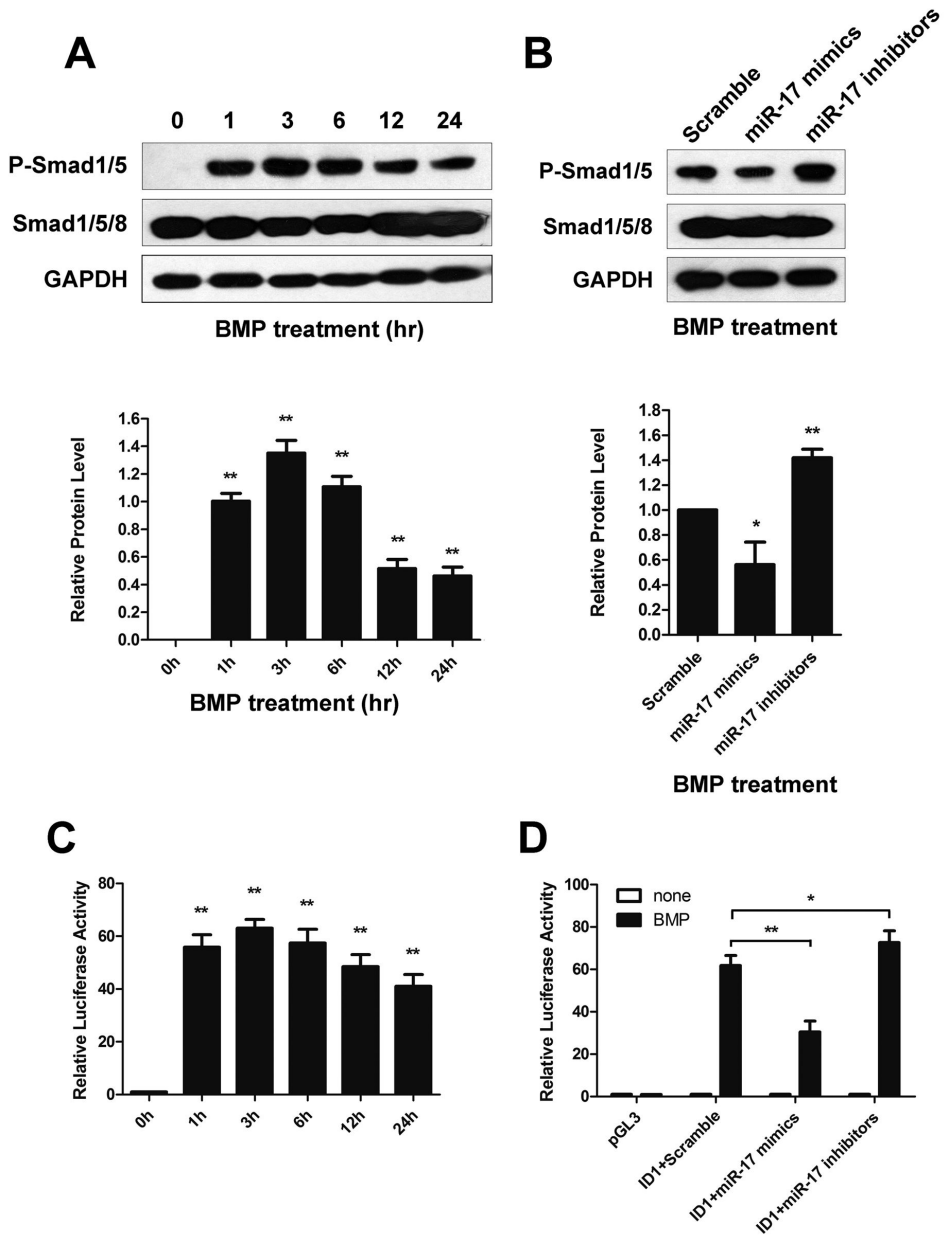
Effects of miR-17 family in BMP2 mediated p-Smad induction. (**A**) Time course of p-Smad protein levels in primary cortical neurons under BMP2 (10 ng/ml) treatment: representative Western blot (upper panel) and quantitative analysis of p-Smad level normalized by Smad (bottom panel). **p < 0.01, vs 0 h. (**B**) Protein analysis of p-Smad in different groups of primary culture at 3 h after BMP2 stimulation: representative Western blot (upper panel) and quantitative analysis of p-Smad level normalized by Smad (bottom panel). *p < 0.05, **p < 0.01, vs Scramble. (**C**) Time course of luciferase reporter activity in SH-SY5Y cells under BMP2 (10 ng/ml) treatment. **p < 0.01, vs 0 h. (**D**) Luciferase reporter activity of different groups of SH-SY5Y cells at 3 h after BMP2 stimulation. *p < 0.05, **p < 0.01. Data shown are the mean ± SD for three independent experiments.

Since BMP signal was transduced by p-Smad, we further investigated the effect of miR-17 on BMP-induced p-Smad level. We demonstrated that BMP2 induced the p-Smad1/5 level in a time-course manner, which reached peak at 3 h and down-regulated during 12-24 h after BMP2 stimulation (Figure 4A). Then we further analyzed the p-Smad level at 3 h after BMP2 treatment with gain and loss of miR-17. It is showed that miR-17 mimics reduced p-Smad level (0.56±0.18 fold) while miR-17 inhibitor significantly increased p-Smad level (1.42±0.07 fold) compared to control (Figure 4B). In addition, we conducted luciferase assay and found that BMP2 activation significantly increased the expression of reporter gene driven by the promoter of ID1 in a time-course manner, which is similar to the change of p-Smad level (Figure 4C). Furthermore, pretreatment with miR-17 mimics blocked such increase (reduced to 30.42±5.21 fold), while miR-17 inhibitor significantly strengthened the increase of reporter gene (increased to 72.64±5.56 fold) compared with scrambles (63±3.48 fold, Figure 4D). These results indicated that miR-17 family may regulate Smad activity after BMP2 stimulation via the target protein BMPRII.

### Function of the negative feedback loop of BMP-miR-17 family-BMPRII in neuron

**Figure 5:**
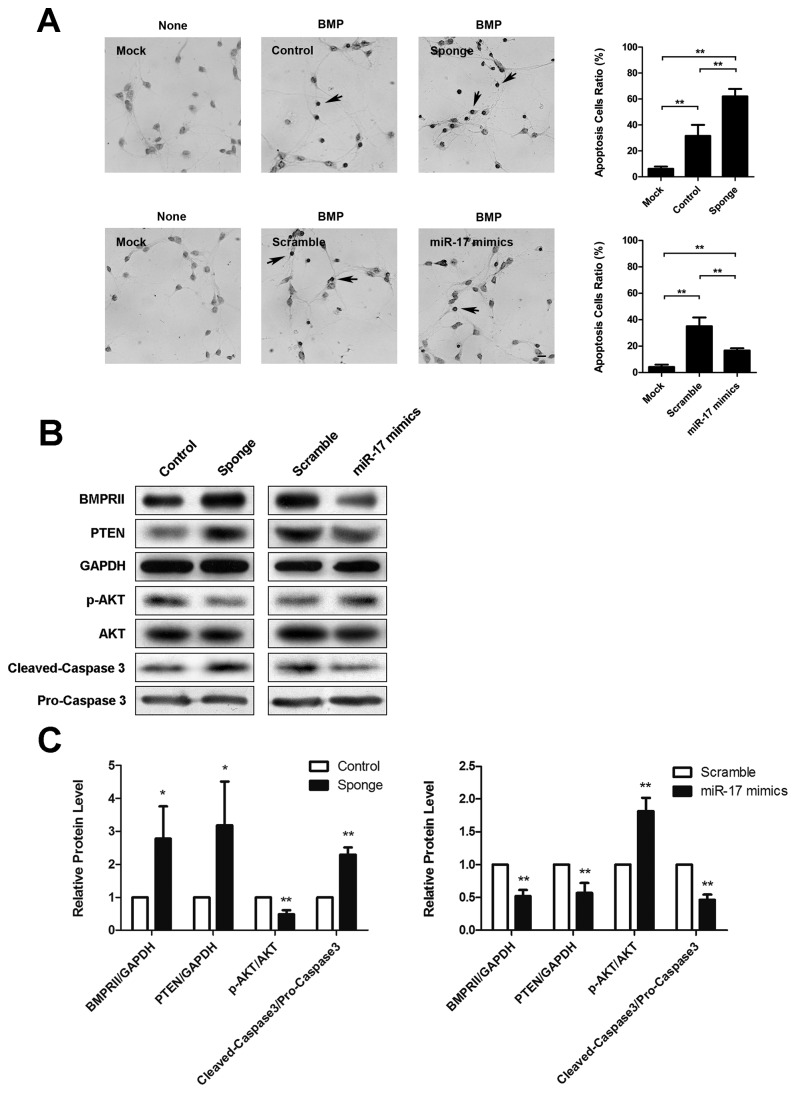
Functional analysis of BMP-miR-17 family-BMPRII negative feedback loop in neuron apoptosis. (**A**) TUNEL assay and quantification in different groups of primary cortical neurons. Cells were transfected with miR-17 family sponge or miR-17 mimics for 12 h, followed with or without BMP2 (50 ng/ml) treatment for 5 days (left panels, scale bar = 20 μm, arrows: TUNEL positive cells). Quantitative analysis of apoptotic cell ratios is shown (right panels). *p< 0.05, **p < 0.01, vs Mock. (**B**) Western blot of BMPRII, PTEN, p-AKT and cleaved-caspase 3 levels in primary cortical neuron tranfected with miR-17 family sponge or miR-17 mimics followed with BMP2 (50ng/ml) treatment for 6h. (**C**-**D**) Quantitative analysis of BMPRII, PTEN, p-AKT and cleaved-caspase 3 levels in differently treated neurons. *p< 0.05, **p < 0.01, vs Control/Scramble. Data shown are the mean ± SD for three independent experiments.

The regulation of BMP pathway in nervous system is crucial since aberrant activation of BMP signaling leads to cellular dysfunction such as apoptosis [12]. In the present study, we conducted TUNEL assay and demonstrated that the apoptotic ratio of primary cortical neurons showed a remarkable increase upon the treatment of high dose of BMP2 (50 ng/ml) (Figure 5A)

When we blocked the negative feedback loop via miR-17 family sponge which increased BMPRII level (Figure 5B), we found the neuronal apoptosis was increased (from ~32% to ~62%) (Figure 5A). On the contrary, over-expression of miR-17 strengthened the negative feedback loop via targeting BMPRII (Figure 5B), which significantly decrease the signal of TUNEL staining in primary neurons (from ~35% to ~17%) (Figure 5A). Meanwhile, protein level analysis showed that apoptotic related genes including PTEN, p-AKT and cleaved-caspase 3 changed consistently when the neuronal apoptosis was augmented by miR-17 family sponge. When we over-expressed miR-17, the expression levels of these apoptotic related genes changed in the opposite direction indicating that cellular apoptosis was repressed (Figure 5C) [30–33].

**Figure 6:**
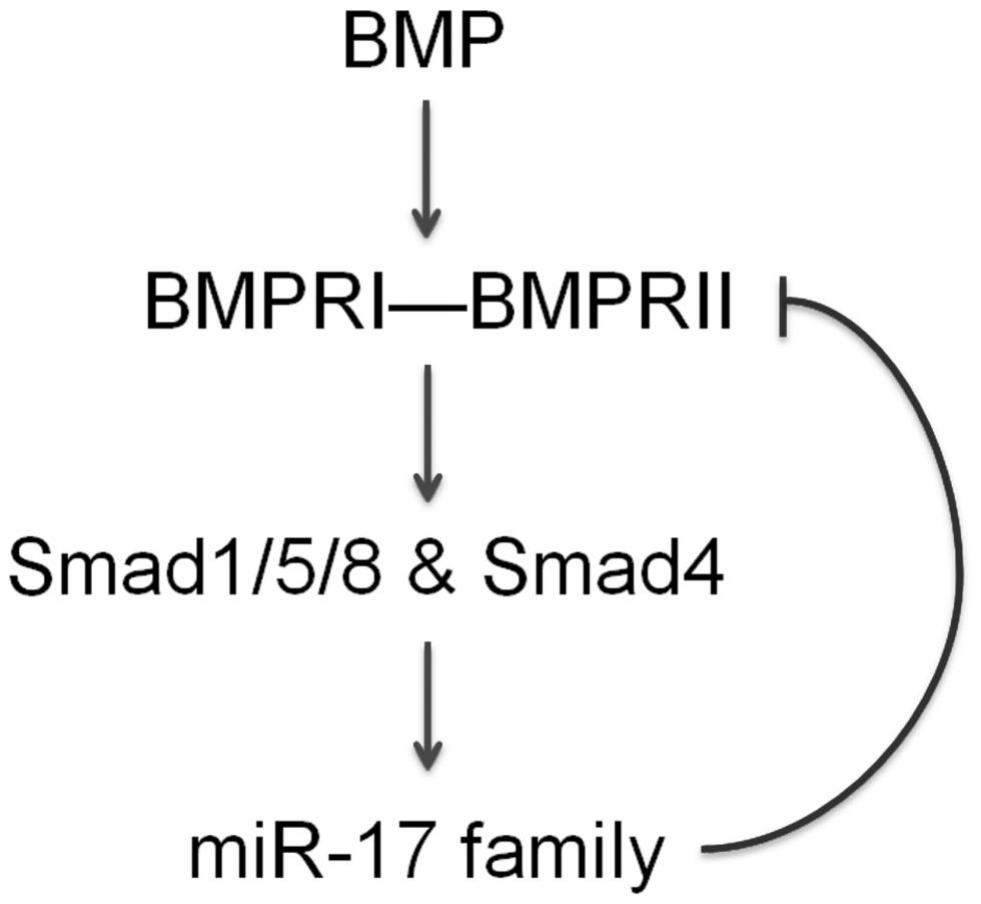
Schematic diagram of a negative regulatory loop of BMP-miR-17 family-BMPRII. BMPs activate Smads via BMPRI-II complex, leading to the increase of miR-17 family, which represses BMPRII expression to prevent aberrant activation of this signaling pathway.

In conclusion, these data suggest that miR-17 family play an important role in a negative feedback loop of BMP signaling pathway (Figure 6), which helps to maintain cellular homeostasis and prevent apoptosis when BMP signaling is aberrant.

## Discussion

BMP signaling pathway is involved in many essential biological processes, in which it regulates gene expression through the activation of Smads. In central nervous system, BMP signaling is involved in cell differentiation, maturation and even apoptosis. Since this signaling is indispensable under normal condition, it is important for us to elucidate the mechanism under the regulation of this pathway.

In our present study, we demonstrated that the transcription of miR-17-92 and miR-106b-25 cluster increased after BMP2 stimulation in a Smad-dependent manner in primary neurons. Further investigation also showed that Smad complex directly bound to miR-106b-25 chromatin. Bioinformatics approaches revealed a conserved Smad-binding element (SBE) in miR-106b-25 promoter. Besides, a Smad recognition element was also presented in the miR-17-92 promoter based on the previous report [22]. These data suggest that BMP signaling directly increase the transcription of miR-17-92 and miR-106b-25 cluster via activation of R-Smad. Moreover, the Smad-binding element presented in the promoter of miR-106b-25 cluster is highly conserved in many species indicating that modulation of miR-106b-25 cluster by BMP signaling is universal (Figure S1).

It is reported that miR-17 and miR-20a can repress the expression of BMPRII in endothelial cells [21]. Our results provided evidences that miR-17 and miR-93, another member of miR-17 family can also repress BMPRII translation in primary neurons. This regulation is important since it is reported that miR-17 family is highly expressed in the early development of nervous system [34]. The high level of miR-17 family may target BMPRII leading to the down-regulation of BMP signaling, while the repression of BMP signaling has been prove to facilitate neurogenesis [8]. Furthermore, the targeted sequence exists in the 3’-UTR of BMPRII mRNA is highly conserved in many species. These data indicate that the repression of BMPRII expression by miR-17 family is also conserved, which bears crucial physiological functions.

There is a report that miR-17/20a can negatively modulate the positive auto-regulatory loop of E2F1–3 to control the concentration of related transcriptional factors [35], which suggests the role of miR-17 family in maintaining cell balance. Our data has demonstrated the relation between BMP, miR-17 family and BMPRII, in which up-regulated miR-17 family made great contribution to BMP2 mediated BMPRII repression, suggesting an important role of miR-17 family in the negative feedback loop of BMP signaling in primary neuron. There has been a study indicating a regulatory loop between BMP4 and miR-302 in pulmonary artery smooth muscle cells, in which BMP4 strengthens its signaling through the inhibition of miR-302 that targets BMPRII [26]. They have demonstrated that the level of BMPRII is increased upon BMP4 treatment, which seems to be contradictory to our findings. However, such regulatory effect is applicable in the model with high basal level of miR-302 enough to repress BMPRII expression, which may not be the exact situation in primary neurons. In our model, we provided strong evidence that BMP2 increased the level of miR-17 family, which further repressed BMPRII expression in neuron. Therefore, it becomes a negative feedback loop which stabilizes the BMP signaling in cells.

The physiological function of this negative feedback loop is thought to maintain cellular homeostasis by preventing the over-stimulation of BMP signaling since aberrant activation of BMP signaling may inhibit the cell proliferation and cause apoptosis [12]. Our results demonstrated that high concentration of BMP2 induced apoptosis of primary neurons. When we broke the negative feedback loop by blocking the function of miR-17 family with sponge, the apoptotic ratio significantly increased. Contrarily, when we over-expressed miR-17 in primary neurons that strengthened the feedback effect, those cells displayed increased resistance to BMP-induced apoptosis. These results support the notion that miR-17 family negatively regulates the BMP signaling via targeting BMPRII in the presence of high dose of BMP, which is beneficial to cell homeostasis.

Taken together, our data provide strong evidences that up-regulated miR-17 family represses BMPRII expression in neurons after BMP stimulation, which consist a negative feedback loop that plays a crucial role in the maintaining of cellular homeostasis and prevent apoptosis upon the overdose of BMP signaling.

## Supporting Information

Figure S1 **SBE in the promoter region of miR-106b-25 cluster locus among mammals.**
(TIF)
