# Supplementary figures and images for: Role of miR-17 Family in the Negative Feedback Loop of Bone Morphogenetic Protein Signaling in Neuron
Source: PLoS One. 2013 Dec 11;8(12):e83067. doi: 10.1371/journal.pone.0083067 (PMC3859655; doi:10.1371/journal.pone.0083067)

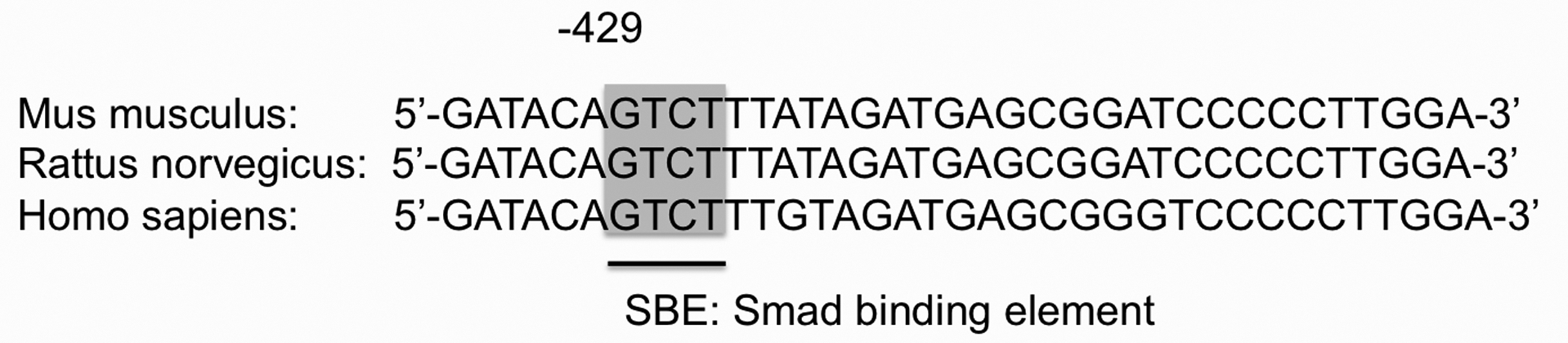

Supplement: Figure S1 — SBE in the promoter region of miR-106b-25 cluster locus among mammals. (TIF) [file pone.0083067.s001.tif]
